# *Notes from the Field:* Blastomycosis Cases Occurring Outside of Regions with Known Endemicity — New York, 2007–2017

**DOI:** 10.15585/mmwr.mm6738a8

**Published:** 2018-09-28

**Authors:** Robert McDonald, Elizabeth Dufort, Brendan R. Jackson, Ellis H. Tobin, Alexandra Newman, Kaitlin Benedict, Debra Blog

**Affiliations:** ^1^Epidemic Intelligence Service, CDC; ^2^New York State Department of Health, Albany, New York; ^3^Division of Foodborne, Waterborne, and Environmental Diseases, National Center for Emerging and Zoonotic Infectious Diseases, CDC; ^4^Upstate Infectious Diseases Associates, Albany Medical Center, Albany, New York.

In October 2017, the New York State Department of Health was alerted by Albany-area infectious disease physicians about local cases of blastomycosis, including multiple severe infections, in the state health department’s Capital District, an area where *Blastomyces* spp. fungi are not considered endemic. The majority of patients reported no travel to regions where blastomycosis is known to be endemic, prompting a state investigation of the disease. Blastomycosis is reportable in only five states (Arkansas, Louisiana, Michigan, Minnesota, and Wisconsin); it is not reportable in New York. To evaluate New York blastomycosis trends, statewide health care data were reviewed for the period 2007–June 2017, and incidence in one county in the Capital District was found to be particularly high. Although not a reportable disease, as an emerging infectious disease in New York, suspected blastomycosis should be reported to local health departments where patients reside.

Blastomycosis is an uncommon and underdiagnosed disease caused by inhalation of *Blastomyces* spp. fungi, which grow in moist soil and organic matter. Based on reports of animal and human cases, *Blastomyces* spp. are thought to be endemic in areas of North America along the Great Lakes and the Mississippi, Ohio, and Saint Lawrence River valleys (*1*). Unlike the similar fungal diseases coccidioidomycosis and histoplasmosis, a skin test has not been available to assess geographic distribution of exposure. Data regarding blastomycosis in New York are limited, with reports of canine infections suggesting endemicity along the Saint Lawrence River on the New York-Canada border (*2*). Outdoor exposures and proximity to waterways have been associated with the disease; however, little about its ecology and epidemiology is known.

Pneumonia is the most common manifestation of blastomycosis. Approximately half of blastomycosis infections can be asymptomatic; however, infection can lead to severe and fatal disease, often from respiratory failure. Disseminated infection can involve any organ, often including cutaneous abscesses and osteomyelitis, and is frequently accompanied by fever, weight loss, and night sweats. Blastomycosis is treated with antifungal medications, typically itraconazole or another azole for mild or moderate disease and lipid formulations of amphotericin B for severe disease. Delays in diagnosis of more than 1 month have been observed in >40% of patients (*3*), suggesting that a diagnosis of blastomycosis is often not considered until after other treatments have failed. Blastomycosis diagnosis can be confirmed by fungal culture, with the optimal specimen source depending on the type of infection. Pulmonary blastomycosis can be detected on sputum and lower respiratory cultures. *Blastomyces* spp. also can be identified on histopathology. Polymerase chain reaction can be used to confirm culture or histopathologic identification and on blood to detect disseminated disease. *Blastomyces* spp. antigen and antibody tests can aid in diagnosis, although clinicians should be aware that these tests have limited sensitivity and can cross-react with *Histoplasma* *capsulatum* and other fungi (*4*). 

A 2012 epidemiologic and ecologic review found that in Illinois and Wisconsin, where blastomycosis is considered endemic, the range of annual incidence was 0.4–2.6 cases per 100,000 population (*1*). To evaluate New York blastomycosis trends, statewide hospital, emergency department, and hospital-associated outpatient *International Classification of Diseases* (ICD) codes from the Statewide Planning and Research Cooperative System data set were reviewed for the period 2007–June 2017. During 2007–2015, blastomycosis ICD codes were identified for an annual mean of 24 (range = 17–30) patients (average annual incidence = 0.1 cases per 100,000 population). In 2016, blastomycosis ICD codes were identified for 59 patients (incidence = 0.2 cases per 100,000). Preliminary data from the first 6 months of 2017 include 25 patients, above the previous annual mean. Incidence in one county along the Mohawk River in the Capital District was particularly high, with a mean of 2.2 cases per 100,000 (range = 0–6.1) during 2007–2016. Travel and exposure information were not available.

Although ICD codes likely involve misclassification, identifying only a small proportion of infections, these data, combined with case reports, indicate that blastomycosis might be endemic in eastern upstate New York. These findings, along with reported cases of blastomycosis in Texas, Kansas, Nebraska (*5*), and Vermont (*6*), highlight limitations of the existing blastomycosis endemic map* and the need for better data.

Active blastomycosis case finding is under way in New York, as is investigation of passively reported cases to assess common exposures, better characterize risk factors, and evaluate a possible common source associated with the high incidence in the county along the Mohawk River. Although blastomycosis is not currently a reportable disease, health care providers and health care facilities should report suspected cases as an emerging infectious disease in New York to local health departments where patients reside. To prevent delays in diagnosis, which can lead to more severe illness and death, clinicians and laboratorians should be aware that blastomycosis can be acquired in areas outside of regions where the disease is considered endemic and to consider the diagnosis in patients with compatible signs and symptoms.

## Footnotes

* https://www.cdc.gov/fungal/diseases/blastomycosis/causes.html.
